# Research progress of radiation esophagitis: A narrative review

**DOI:** 10.1097/MD.0000000000042273

**Published:** 2025-05-09

**Authors:** Qiang Wu, Qiuxia Li, Ruidan Zhang, Yongbin Cui, Jingmin Zou, Jianbo Zhang, Changsheng Ma, Dali Han, Yiru Peng

**Affiliations:** a Shandong Second Medical University, Weifang, Shandong Province, China; b Shandong Cancer Hospital and Institute, Shandong First Medical University and Shandong Academy of Medical Sciences, Jinan, Shandong Province, China; c Department of Journal Center, Shandong Provincal Hospital Affiliated to Shandong First Medical University, Jinan, Shandong Province, China.

**Keywords:** prediction model, prevention and treatment, radiation esophagitis, risk factors

## Abstract

Radiation esophagitis (RE) is an adverse reaction to radiotherapy for thoracic malignancies and a major factor limiting the dosage for thoracic tumors. In cases of RE, mild conditions may involve only discomfort in the throat, severe cases can present with intense pain and significant difficulty swallowing, and may lead to interruptions in radiation therapy, affecting the treatment outcome. However, much of the current research focuses on how to diagnose and treat the disease and needs to address the prevention of RE. In this review, several online databases, including PubMed, Web of Science, Google Scholar, the China National Knowledge Infrastructure, and the Chinese Medical Journal Database, were searched to collect relevant published studies. This review provides updated information on the pathogenesis, diagnosis, risk factors, development of predictive models, prevention, and treatment of RE, aiming to serve as a reference for its diagnosis, treatment, and prevention.

## 1. Introduction

Radiation therapy has become an increasingly important treatment method in tumor treatment. However, radiation esophagitis (RE) remains a common complication of radiotherapy for thoracic malignancies.^[1]^ Up to 42% of patients with lung, breast, esophageal or mediastinal lymphoma experience RE after radiotherapy.^[2]^

RE is one of the dose-limiting toxicities of radiotherapy for thoracic tumors. Symptoms of acute RE typically appear within 2 to 3 weeks of thoracic radiation therapy and can persist for 4 weeks after completion of radiation therapy.^[3]^ At a radiation dose of 10 to 20 Gy, normal esophageal mucosa may experience congestion, edema, and dysphagia; however, when the dose increases to 30 to 40 Gy, mucosal congestion worsens, possibly causing local pain or a burning sensation behind the sternum, especially when eating. In very rare cases, it can lead to severe complications such as esophageal perforation, bleeding, or the formation of a fistula between the esophagus and trachea.^[4–6]^ The severity of RE symptoms can be classified using the National Cancer Institute (NCI) Common Terminology Criteria for Adverse Events (CTCAE) version 5.0,^[7]^ and the Radiation Therapy Oncology Group (RTOG)^[8]^ toxicity criteria, as shown in Table 1.

**Table 1:** Grading criteria for RE.

Grading	RTOG		CTCAE v5.0
	Acute	Chronic	
0	No adverse reaction	No adverse reaction	No adverse reaction
1	Mild dysphagia or pain, need to be relieved by topical anesthetic or enter a half-stream diet	Mild fibrosis or difficulty swallowing solid food; no swallowing pain	Asymptomatic; only found by clinical examination and diagnosis;no treatment required
2	Moderate dysphagia or pain in swallowing, anesthetic analgesia or liquid diet	Cannot enter solid food or semisolid food normally or may have an indication of expansion	Difficulty in eating or swallowing; need oral nutrition
3	Severe dysphagia or pain in swallowing, with dehydration or weight loss > 15%, need to be supplemented by nasal feeding or intravenous infusion	Severe fibrosis or only flow into food or swallow pain or need to dilate	Severe difficulty in eating or swallowing; requires nasal feeding, total parenteral nutrition or hospitalization
4	Complete obstruction, ulcer, perforation or fistula formation	Necrosis or perforation or fistula	Life-threatening; requiring emergency surgical treatment
5			Death

CTCAE = common terminology criteria for adverse events, RE = radiation esophagitis, RTOG = radiation therapy oncology group.

## 2. Methods

We conducted a literature search on RE using PubMed, Web of Science, Google Scholar, the China National Knowledge Infrastructure, and the Chinese Medical Journal Database. The search terms included “RE,” “diagnosis,” “influencing factors,” “predictive models,” “treatment,” and “prevention.” Relevant literature was screened through manual review. The search timeframe spanned from the inception of each database to 2024. Inclusion criteria encompassed randomized controlled trials, basic research, review articles, systematic reviews, and case reports, with priority given to newer publications or those with higher impact factors for overlapping studies. Exclusion criteria involved literature unrelated to the research topic, duplicate publications, controversial findings, preclinical studies, and conference abstracts. The study was independently conducted by 2 researchers, who evaluated the sources and content of the literature. Ultimately, 90 articles were included in the analysis.

## 3. The mechanism of occurrence of RE

Radiation therapy is a common treatment method for thoracic tumors that effectively alleviates symptoms related to patients with tumors. Owing to the current limitations of treatment technologies, radiation inevitably causes a certain degree of damage to the surrounding normal tissues while killing tumor cells. The ionizing radiation produced by radiation therapy leads to direct DNA damage within the treatment area, including double-strand breaks, single-strand breaks, and elimination of bases at certain sites, thereby inducing programmed cell death.^[9]^ Radiation therapy can also indirectly damage DNA (via reactive oxygen species), triggering a series of events that may lead to cell death. Radiation also breaks down water molecules in esophageal tissues to form hydroxyl radicals, inducing oxidative stress, thereby causing damage to normal esophageal tissues.^[10]^

Radiation-induced damage can activate stress-induced signaling pathways and release proinflammatory cytokines.^[11]^ The primary effector cells are monocytes and macrophages, which when excessively activated by radiation stimulation, release a large amount of inflammatory mediators, such as IL-6, IL-10, and TNF-α, leading to pathological damage. Particularly, the squamous epithelium of the esophagus, which is relatively sensitive to radioactive materials,^[12]^ can cause a slowdown in esophageal peristalsis when damaged. This slowdown in peristalsis extends the time harmful substances pass through the esophagus, exacerbating damage. Additionally, radiation therapy can cause bone marrow suppression in the body and reduce immunity, thereby leading to esophageal infections and esophagitis.^[13]^

## 4. Diagnosis of RE

Clinically, RE is primarily diagnosed by combining a patient’s history of radiation therapy and excluding other diseases based on symptoms. Since early stage RE is sterile inflammation, laboratory tests such as white blood cell count, C-reactive protein, and procalcitonin often do not show an increase.^[14]^ Esophagography with barium can assist in the diagnosis of RE. In early symptomatic cases, barium meal can cause weakened esophageal peristaltic waves, beak-like narrowing, and esophageal ulcers. Esophageal strictures can be observed.^[15–17]^ Esophagoscopy is also helpful for the diagnosis and treatment of RE and can help differentiate between radiation damage and infectious esophagitis.^[18]^ Endoscopic findings vary, including erythema, erosion, mucosal detachment, ulcers, and bleeding. Esophagoscopy not only allows direct observation of the lesion but also enables biopsy of the esophageal tissue to assess the severity of esophagitis. Tissue sampling is crucial for ruling out recurrent malignant tumors.^[19]^

The pathological manifestations of RE include esophageal congestion, edema, erosion, inflammatory exudative changes, and ulcers. Mouse experiments have shown^[10]^ that 7 days after irradiation, pathological changes occur in the mucosal layer of the esophagus, with thickening of the stratified squamous epithelium and involvement of the submucosal layer; by day 14, there was more severe damage to the squamous epithelium and spines of the mucosal epithelium; by day 21, necrosis and detachment of the mucosal epithelium occurred, with edema or vacuolation in the basal cell layer and moderate inflammatory cell infiltration in the lamina propria; by day 28, there was severe necrosis and detachment of the mucosal epithelium, with multiple vacuoles appearing in the basal cell layer, and some cases showed ulcerative lesions. Typical pathological characteristics of late RE^[18]^ include chronic inflammation, fibrosis, and dysplasia.

## 5. Risk factors of RE

Despite numerous studies exploring these risk factors, our ability to identify RE risk factors remains limited. Some of the identified risk factors appear to be contradictory. Generally, risk factors for RE can be categorized into 4 main areas: patient-related factors, tumor factors, treatment-related factors, and other factors.

### 5.1. Patient factors

The general condition, nutritional status, and underlying diseases of the patients are closely related to RE. Some reports suggest that the sex and age of patients with thoracic tumors are related to RE,^[20]^ but most studies have not found sex and age to be risk factors for RE.^[21–23]^ Regarding RE, Komiya^[24]^ suggested that there is a certain relationship with ethnicity, but Laucis et al^[25]^ did not find a correlation between esophagitis and race. therefore, the relationship between RE and ethnicity requires further research.

Studies have found that patients with hypertension and diabetes are more susceptible to RE. This may be related to the decreased venous elasticity caused by hypertension, thickening of the small arterial walls, and sparseness of the capillary network,^[26]^ which leads to an inadequate blood supply to the local tissue after radiation damage, resulting in edema and reduced repair capacity. Additionally, hypertension may stimulate the release of inflammatory factors, triggering a vascular response, thereby causing local ischemia, hypoxia, and circulatory issues in the esophagus, as well as tissue edema. This not only reduces the tissue’s self-repair capacity but also increases the risk of RE.^[27]^ The persistent high blood sugar state in diabetic patients lowers the body’s immunity and promotes radiation-induced inflammation. This state may induce a series of issues, such as inflammatory responses, immune dysfunction, oxidative stress, damage to the microvascular endothelium, and microvascular occlusion.These factors lead to fibrinoid and steatosis of the blood vessel wall, increase the permeability of the blood vessel wall, aggravate the inflammatory response of esophageal tissue, and increase the risk of esophagitis.^[28,29]^ Studies show that^[30]^ both diabetes and hypertension can reduce a patient’s immunity and tissue repair capability, exacerbating the extent of inflammatory damage and thus increasing the risk of esophagitis.

Malnutrition and performance status are also risk factors for RE, Dong et al^[30]^ found that the initial nutritional status is closely related to the occurrence of ≥ grade 2 RE in patients with esophageal cancer receiving radiation therapy, with 41% of patients with severe malnutrition experiencing ≥ grade 2 RE. Daniel et al^[31]^ analyzed the risk factors for lung cancer RE and found thatthe initial nutritional status is a risk factor for RE.

### 5.2. Tumor factors

The location of the tumor in the thorax, the length of the irradiated esophagus, the stage of the tumor, and whether the tumor is accompanied by infection are risk factors for RE.

The location of a tumor are closely related to RE. Xia Xinye et al found that patients with central lung cancer were more likely to develop RE, which is a risk factor for RE in patients with NSCLC receiving 3D-CRT treatment.^[32]^ This may be related to the proximity of the esophagus to the lesion in patients with central lung cancer, making it difficult to avoid irradiation of the esophagus during target area delineation. Sadia et al^[33]^ found that left-sided breast cancer is more likely to cause esophageal cancer than is right-sided breast cancer.

Bhaskaran et al^[34]^ also found that the incidence of grade 2 esophagitis in patients with left-sided breast cancer (11/20, 55%) was higher than that in those with right-sided breast cancer (9/20, 45%). The reason may be that the esophagus is located on the left side of the midline, which causes the esophagus to receive a higher dose during radiotherapy for left breast cancer than for right breast cancer. Ye et al^[23]^ found that upper esophageal cancer is more likely to cause RE, which may be related to the longitudinal course of sensory nerves.^[35,36]^ The distribution of sensory axons in the upper part of esophageal cancer is different from that in the lower part, making the upper part more sensitive to damage.

Lymph node involvement is associated with a higher incidence of RE. Wang et al^[37]^ found that for breast cancer patients undergoing postoperative radiotherapy, irradiation to the internal mammary lymph nodes increased the incidence of RE of grade ≥ 2, because irradiation to the internal mammary lymph nodes resulted in increased esophageal radiation dose and increased the incidence of RE.The length of the esophagus irradiated in the treatment area is also a risk factor for RE,the longer the length of the esophagus in the treatment area, the greater the range and dose of irradiation to the esophagus, making RE more likely to occur.^[18,38]^

Inflammatory factors are also risk factors for esophagitis. Virchow first proposed the connection between cancer and inflammation in 1863, and increasing evidence suggests^[39,40]^ that inflammation promotes cancer progression by altering the tumor microenvironment. Chronic inflammation triggers molecular cascading reactions within tumor cells, thereby promoting tumor invasion and evasion of immune cells. The Systemic Inflammatory Response Index (SIRI) and platelet-albumin ratio (PAR) are significantly associated with ≥ grade 2RE and have been used to predict the incidence of RE in patients with small cell lung cancer undergoing radiochemotherapy.^[41]^ The neutrophil-to-lymphocyte ratio (NLR) and platelet-to-lymphocyte ratio (PLR) are significantly associated with ≥ grade 2 RE and have been used to predict the incidence of RE in patients with locally advanced esophageal squamous cell carcinoma undergoing radical chemoradiotherapy.^[22]^

### 5.3. Treatment factors

Dose factors are significant risk factors for toxicity of esophagitis,^[13,42]^ with symptoms of acute esophagitis typically appearing when the radiation dose reaches 18.0 to 21.0 Gy. Higher doses of radiation therapy are more likely to induce RE, and as radiation therapy progresses, damage to the esophageal nerves and muscles, leading to weakened esophageal motility, also increases the probability of esophagitis.^[43]^ Zahra et al^[44]^ studied radiotherapy for breast cancer and found that limiting esophageal doses to < 13 Gy helped reduce the frequency and severity of grade 2 or higher toxicities. Daniel et al^[31]^ study analyzing lung cancer RE found that the mean esophageal dose (MED) and minimum dose to the 2 cc of esophagus receiving the highest dose (D2cc) were significantly associated with grade 2 + and grade 3 + esophagitis. Yu et al^[22]^ studied esophageal squamous cell carcinoma and found that patients receiving radiation doses > 61.5 Gy were more likely to develop Grade ≥ 2 RE than patients receiving radiation doses ≤ 61.5 Gy.The RTOG0617 study compared the treatment effects of standard dose (60 Gy) and high-dose (74 Gy) radiation therapy with concurrent chemotherapy, and found that the occurrence of grade 3 RE was lower in the 60 Gy group than in the 74 Gy group. In the 60 Gy group, 3% and 5% of patients experienced treatment-related ≥ grade 3 dysphagia and esophagitis, respectively, whereas in the 74 Gy group, these percentages were 12% and 17%.^[45]^ Wang et al^[37]^ found that RV25 (the relative percentage volume receiving 25 Gy) and AV35 (the absolute percentage volume receiving 35 Gy) are the best dose-volume predictors for ≥ grade 2 RE, further illustrating that dose factors are important risk factors for RE.

Concurrent chemoradiotherapy increases the likelihood of RE development. Compared with sequential chemoradiotherapy, concurrent chemoradiotherapy improves the 2-year survival rate by 10% in patients with locally advanced NSCLC. However, concurrent chemoradiotherapy also approximately quintuples the risk of acute RE.^[46]^ Zhang et al^[47]^ found a similar situation in their research, which showed that concurrent chemoradiotherapy significantly increased grade 3 to 4 adverse reactions from 4% to 18% compared with sequential chemoradiotherapy. This analysis suggests that chemotherapy drugs can alter the proliferation rate of connective tissue cells in the lamina propria, leading to increased vascular permeability and inflammatory infiltration, delayed injury repair, and increased risk of esophagitis in patients.^[48]^ However, there are fewer studies have indicated that RE is not related with concurrent chemoradiotherapy.^[38]^

The likelihood of developing RE varies with different radiation therapy techniques. A study by Li et al^[49]^ comparing inoperable stage I Non-Small Cell Lung Cancer (NSCLC) treated with SBRT and 3D-CRT found that the risk of adverse reactions, such as dyspnea, radiation pneumonia, and esophagitis, was significantly lower in the SBRT group than in the 3D-CRT group. Lin et al^[50]^ found that intensity modulated radiotherapy can reduce the frequency and severity of pain events in RE caused by NSCLC.Yaney et al^[51]^ indicated that patients treated with IMRT had a significantly higher incidence of grade 2 RE compared to those receiving 3D conformal radiation therapy, proton therapy, and carbon ion therapy, with their physical advantage of the Bragg peak, representing technological improvements over photon conformal therapy. Multiple studies^[52–54]^ have shown that proton therapy and carbon ion therapy yield better results and have fewer side effects than photon therapy, thereby reducing the occurrence of adverse reactions.

Different segmentation methods have different probabilities of causing RE.The standard treatment for patients with locally advanced NSCLC is radical concurrent chemoradiotherapy (CCRT); accelerated hyperfractionation radiotherapy (AHRT) improves local control and overall survival. However, compared to traditional segmentation processing,^[55,56]^ AHRT increases the incidence of RE.

### 5.4. Other influencing factors

Single nucleotide polymorphisms (SNPs) have been widely studied in various cancer types because of their involvement in mediating damage and stress responses induced by radiation toxicity. He et al^[57]^ found that the XRCC5 allele increases the risk of severe RE, and amplification of the Zinc Finger Protein 217 gene is associated with an increased risk of severe (grade 3 or above) pneumonia and esophagitis. Aguado-Barrera et al^[58]^ also confirmed the relationship between SNPs and adverse reactions in NSCLC patients receiving radiation therapy, and found that rs4772468 of the FGF14 gene could increase the risk of RE. Yuan et al^[59]^ discovered that genetic variations in the transforming growth factor-beta1 (TGFβ1) pathway are associated with adverse thoracic radiation therapy reactions in the lung, esophagus, and pericardium. Carriers of the TGFβ1-509 T allele had significantly less severe RE (*P* = .019) and a lower average grade of thoracic radiation therapy adverse reactions (*P* = .009) than patients with the TGFβ1CC genotype.

Furthermore, the distribution characteristics of the gut microbiota were significantly correlated with RE severity. Studies have shown^[59]^ that patients with a higher abundance of Clostridium before radiation therapy are more likely to develop severe acute RE, whereas patients with a higher abundance of Klebsiella, Roseburia, and Veillonella genera are more likely to develop mild acute RE.

## 6. The development of prediction model

In recent years, with the progress in RE research, the understanding of RE has rapidly increased. Various predictive models have been constructed to better predict and manage RE based on risk factors.

Various models for predicting RE were analyzed.^[4,23,24,41,50,60–64]^ There are several common ways to build prediction models: analyzing patients’ baseline characteristics (such as gender, age, health status, etc) and treatment-related parameters (such as radiation dose, the size and location of the treatment area, etc) to establish risk prediction models; using dose-volume histogram (DVH) analysis techniques to assess the distribution of radiation doses in different volumes of the esophagus, thereby predicting the risk of esophagitis. This method can more accurately describe the radiation dose received by the esophagus, providing important quantitative indicators for risk assessment, and exploring biomarkers related to the risk of radiation-induced esophagitis to predict the occurrence of RE, such as inflammatory factors and DNA damage markers in the blood. Identifying these biomarkers helps to understand the biological mechanisms of radiation-induced esophagitis, offering a new perspective for risk prediction, utilizing artificial intelligence (AI) and machine learning technologies to analyze a large amount of clinical data and treatment parameters, and establishing more precise predictive models. These models can handle complex data relationships, improve the accuracy and efficiency of predictions, and study how individual genetic differences affect the sensitivity to radiation, thereby predicting the risk of esophagitis. This study hopes to achieve more personalized risk assessments or utilize several of the above factors to construct predictive models.

The goal of these predictive models is to provide doctors with a tool for predicting RE, enabling better assessment and management of radiation therapy treatment plans. Ideally, they can assist physicians in customizing treatments and reducing the risk of complications while maintaining therapeutic efficacy. However, further exploration of the integration of different models is required, and validation and optimization in a broader range of clinical trials are necessary to ensure their accuracy and practicality.

## 7. Prevention and treatment of RE

In patients with esophageal cancer,RE often leads to anxiety and fear. Patients might believe that their condition is worsening or that the treatment is ineffective; in some cases, a few may even lose confidence in their treatment. However, it’s important to recognize that RE is both preventable and treatable.

### 7.1. Prevention of RE

Maintaining physical and mental health not only reduces the occurrence of RE but can also slow down the progression of tumors to some extent. It is important to cultivate healthy lifestyle habits, such as avoiding smoking and alcohol consumption; engaging in moderate exercise; maintaining a balanced and nutritious diet; avoiding spicy, hot, and rough foods; drinking a few sips of water after eating to flush residues from the esophageal wall into the stomach, thus keeping the esophagus clean. Depending on the patient’s general condition and disease status, especially in patients with high-risk factors, it is important to control blood sugar, blood pressure, and other indicators. Appropriate radiation therapy modalities, doses, and fractionation schemes should be selected. For patients who require both radiation and chemotherapy, the risks and benefits of synchronous or sequential radiation and chemotherapy should be evaluated to choose the appropriate combined modality therapy.

Various pharmacological trials have been conducted to investigate the prevention of RE. Glutamine has frequently been studied for the prevention of RE. Prophylactic oral supplementation with glutamine can reduce the incidence and severity of both acute and late RE and improve patients’ quality of life.^[65–67]^ Amifostine is another drug that has been extensively studied for the prevention of RE. Its active metabolite, WR-1065, can scavenge free radicals produced by ionizing radiation, thereby reducing the occurrence of RE.^[68]^ However, some studies indicate^[69–71]^ that amifostine pretreatment shows a trend towards reducing the severity of esophagitis associated with concurrent chemoradiotherapy, but the difference was not statistically significant. Moreover, it can cause adverse reactions, such as nausea, vomiting, cardiotoxicity, and myelosuppression; therefore, the choice of drug should be made with caution.

Moreover, Epigallocatechin-3-gallate (EGCG) has been shown to prevent and treat acute RE.^[72]^ The Modified Zhuye Shigao Decoction has been found to prevent RE by inhibiting the production and release of the inflammatory cytokines TNF-α, IL-1β, and IL-8.^[73]^ Soy isoflavones have been demonstrated to have radioprotective effects, which can mitigate the early and late effects of radiation damage in the esophageal tissue.^[74]^ However, such studies are relatively few, and further research is required to substantiate these findings.

### 7.2. Treatment of RE

The treatment approach for RE is symptomatic, with clinical treatments primarily focusing on astringent actions, anti-inflammatory effects, protection of the repair processes of the esophageal mucosa, pain relief, and nutritional support. Therapeutic medications included anesthetics, mucosal protectants, antibiotics, corticosteroids, proton pump inhibitors, vitamins, and other compound preparations.

For mild to moderate dysphagia and pain during swallowing, the following medications or combinations thereof can be applied^[13,18,75–78]^: oral administration of mucosal protectants such as aluminum hydroxide, magnesium carbonate, sucralfate, and montmorillonite powder; medications such as Kangfuxin solution, vitamin B12, and recombinant macrophage colony-stimulating factor to promote mucosal repair; lidocaine or various oral solutions made primarily from lidocaine to alleviate pain; and oral administration of aminoglycoside antibiotics such as gentamicin and corticosteroids such as dexamethasone to prevent and control inflammatory responses and reduce edema.

For moderate-to-severe dysphagia, pain during swallowing, or those at nutritional risk, nasogastric feeding or a liquid diet should be administered, providing high-energy oral nutritional supplements. If symptoms do not improve, hospital admission may be necessary for intravenous fluid support and pain medication.^[79]^ There is also a viewpoint that suggests pausing radiation therapy.^[1,80,81]^ For patients who have difficulty taking oral medications, transdermal fentanyl patches can be used.^[82]^ For patients with poor gastrointestinal motility, prokinetic drugs such as domperidone can be administered, and for those with esophageal spasms, treatments may include nitrates, calcium channel blockers, and anticholinergic drugs.^[83,84]^ Due to damage to esophageal mucosa, the chance of developing fungal infections increases, especially in patients receiving chemotherapy or steroids, antifungal drugs such as nystatin can be used preventively.^[85]^

When esophageal stenosis, ulcers, perforation, or fistulas occur, esophageal dilation may be necessary to expand the esophagus,^[86]^ and esophageal stents may be used to repair perforations or esophageal fistulas, with some cases requiring multiple surgeries.^[87,88]^ For RE with intractable bleeding, radiofrequency ablation can be considered as a treatment method, with 2 cases of RE with difficult-to-control bleeding achieving good therapeutic outcomes through radiofrequency ablation.^[2]^ Recently, He et al^[11]^ achieved promising results in animal experiments using 3D-printed esophageal stents with decellularized extracellular matrix hydrogel for the treatment of RE, providing a promising strategy for RE treatment.

Traditional Chinese medicine (TCM) is also an important approach for treating esophagitis. TCM does not have a specific term equivalent to “esophagitis,” but based on the clinical manifestations of RE, it can be categorized under conditions such as “nausea,” “noisiness,” “acid reflux,” “chest fullness,” and “acid swallowing.” The principles of TCM treatment for esophagitis initially focused on clearing heat and drying dampness, regulating qi and dispersing stasis, and resolving depression and dispersing masses. In later stages, treatments focus on nourishing yin and moistening dryness, strengthening the spleen and stomach, supplementing qi, warming yang, and converging to promote tissue regeneration.^[89,90]^

## 8. Conclusion and outlook

RE is a common complication of radiation treatment for thoracic tumors. RE typically occurs after conventional fractionated radiation doses of 20 to 30 Gy, leading to symptoms such as dysphagia, odynophagia, and a burning sensation behind the sternum due to esophageal mucosal congestion and edema. If not treated in a timely manner, severe cases may require a pause in radiation therapy, thus delaying cancer treatment opportunities. Early detection and treatment of RE is conducive to the smooth progress of radiotherapy and reduces the incidence of RE. The prediction model of RE has shown great potential in this regard.These models offer new possibilities for customized treatment and are expected to improve treatment outcomes and enhance patients’ quality of life. Future research may focus on improving the accuracy and reliability of these predictive models. With the advancement of AI and big data analysis technologies and the integration of more clinical data, biomarkers, and genetic data, the models will become more complex and precise, providing stronger support for clinical decision-making.

## Author contributions

**Conceptualization:** Yiru Peng.

**Funding acquisition:** Yiru Peng, Changsheng Ma, Dali Han.

**Writing – original draft:** Qiang Wu.

**Writing – review & editing:** Qiuxia Li, Ruidan Zhang, Yongbin Cui, Jingmin Zou, Jianbo Zhang.
